# Posttraumatic stress disorder and associated factors in the aftermath of the 2015 earthquake in Nepal: A systematic review and meta-analysis

**DOI:** 10.1371/journal.pone.0310233

**Published:** 2025-02-03

**Authors:** Prayash Paudel, Asutosh Sah, Anil Khanal

**Affiliations:** 1 Institute of Medicine, Bachelor of Medicine and Bachelor of Surgery, Maharajgunj Medical Campus, Tribhuvan University Teaching Hospital, Kathmandu, Nepal; 2 Department of Public Health, Pokhara University School of Health and Allied Sciences, Pokhara, Nepal; İstanbul Nişantaşı Üniversitesi: Istanbul Nisantasi Universitesi, TÜRKIYE

## Abstract

We aimed to estimate the pooled incidence of posttraumatic stress disorder among survivors after the 2015 earthquake in Nepal based on available literature and highlight the psychological effects of natural disasters that can hamper the recovery process in the aftermath of disaster. The study protocol was registered on PROSPERO with reference number CRD42024576444. Electronic databases such as PubMed and Google Scholar were searched for observational studies in English that assessed posttraumatic stress disorder at least 1 month after the earthquake via a validated tool from April 2015 to August 2024. In addition, references to the included studies were thoroughly searched. High-quality articles were included after the risk of bias assessment. The random-effects model was used to calculate the pooled incidence with a 95% confidence interval along with subgroup analysis. An analysis of 25 studies revealed a pooled incidence of 22.6%, ranging from 17.6 to 27.5%. A high degree of heterogeneity (I^2^ = 97.56%, p<0.001) was observed in the results, with the incidence ranging from 3% to 51%. The subgroup analyses revealed that the incidence of posttraumatic stress disorder after an earthquake varied significantly across studies in terms of the time of assessment, sex, age, marital status, ethnicity, educational level, disease history, bereavement, injury to the body, witnessing death, social support, loss of property and damage to one’s house. However, stratified analyses could not fully explain the heterogeneity in the results. Our analysis revealed a high incidence of posttraumatic stress disorder among survivors of the 2015 earthquake in Nepal. Addressing the psychological well-being of these survivors is essential. Psychosocial counseling becomes pivotal in assisting them in coping with the trauma they have experienced since the earthquake.

## Introduction

One of the most destructive and common natural disasters is an earthquake. They frequently happen suddenly and without warning, negatively affecting a large number of people [1].

Nepal is ranked as the 11th most vulnerable country globally to earthquakes [2]. On April 25, 2015, at 11:55 a.m. (Nepal Standard Time), a catastrophic 7.8-magnitude earthquake with its epicenter in Barpak, Gorkha District, northwest of Kathmandu, struck Nepal [3]. Over 300 aftershocks occurred following the initial earthquake [2]. One of the aftershocks, occurring on May 12, 2015, 17 days later, a 6.8 magnitude aftershock led to additional harm and suffering [3]. It has been more than 80 years since Nepal experienced a seismic shock at this scale [4]. According to the United Nations, the earthquake claimed approximately 8,790 lives and injured 22,300 more people, more than one-third of Nepal’s total population. The World Health Organization (WHO) reported that 20% of individuals experiencing earthquake and other humanitarian crises suffer from psychological distress, whereas a smaller portion (3–4%) experience severe mental disorders [5].

Posttraumatic stress disorder (PTSD) is a serious mental illness that can occur after a traumatic event, including a natural disaster. Characterized by four core symptom clusters—intrusion (e.g., flashbacks and nightmares), avoidance (e.g., steering clear of trauma reminders), negative alterations in cognition and mood (e.g., emotional numbness, guilt, or detachment), and hyperarousal (e.g., heightened startle response or difficulty sleeping) [6]. PTSD has several important implications for those who suffer from this condition. In addition to individual psychological consequences, PTSD also has considerable social and economic burdens, disrupting interpersonal relationships, impairing productivity, and straining healthcare systems, among others [7,8]. In postdisaster settings, such as the 2015 earthquake in Nepal, such an understanding is necessary to shape effective mental health interventions and build resilience among survivors [9].

Research from various countries beyond Nepal has examined the prevalence and effects of PTSD after major earthquakes. A study by Cerdá et al. (2013) reported that the prevalence of PTSD after the earthquake that struck Port-au-Prince on January 12, 2010, was 24.6%[10]. Similarly, research by Wu et al. (2014) reported that 40.1% of survivors of the 2008 Wenchuan earthquake in China experienced PTSD [11]. A systematic review of mental health disorders after the Great East Japan Earthquake in 2011 revealed that the prevalence of PTSD ranged from 10% to 53.5% [12]. These variations are linked to factors such as physical injuries, property loss, family deaths, age, gender, low educational levels, and insufficient social support, all of which are associated with PTSD [13–16].

Nepal’s geographical position makes it the 11th most vulnerable country globally to earthquakes, yet few comprehensive studies have focused on the long-term psychological effects of these natural disasters on its population [2]. The catastrophic earthquake of 2015 and subsequent aftershocks profoundly impacted the country, with survivors not only facing physical and economic losses but also enduring substantial psychological distress [4]. While PTSD is a recognized outcome of natural disasters [17], the unique sociocultural, economic, and infrastructural challenges in Nepal, such as limited mental health resources and social support networks [18], suggest that the incidence and factors influencing PTSD in this context may differ from those in other regions. This further highlights the need for a systematic review and meta-analysis of PTSD, specifically among survivors of earthquakes in Nepal, to inform tailored interventions and resource allocations. Second, the frequent occurrence of earthquakes in Nepal underscores the urgent need for this research to provide actionable insights in preparing for and responding to future disasters to ensure that survivors’ mental health needs are met [5].

Earthquakes cause loss of life and property, disrupt societal structures, and disturb economic stability, creating a ripple effect on mental health and psychosocial functioning. The 2015 Nepal earthquake caused unprecedented destruction, rendering hundreds of thousands of people homeless and seriously affecting livelihoods, which are closely related to mental well-being in resource-limited settings [2]. Damage to homes and loss of property, as highlighted in this meta-analysis, were found to be significant predictors of PTSD, emphasizing the psychological toll of losing one’s shelter and financial security [18]. In addition to material loss, the societal impact of disasters includes weakened social networks, disrupted communities, and reduced access to healthcare services, all of which exacerbate stress and hinder recovery [5]. Furthermore, survivors in economically poor regions, such as Nepal, cannot always afford to rebuild their houses or villages, which exacerbates their mental distress and delays recovery [4]. Addressing postdisaster mental health, therefore, must entail the reconstruction of not only infrastructure but also community cohesion and economic stability to ensure the comprehensive recovery of affected populations. This integrated model also underlines the role that targeted psychosocial interventions and economic support could and should play in disaster preparedness and response.

## Methods

### Study protocol

The study protocol, with well-defined methodology and inclusion criteria, was registered on PROSPERO with reference number CRD42024576444.

### Search strategy

**Fig 1:**
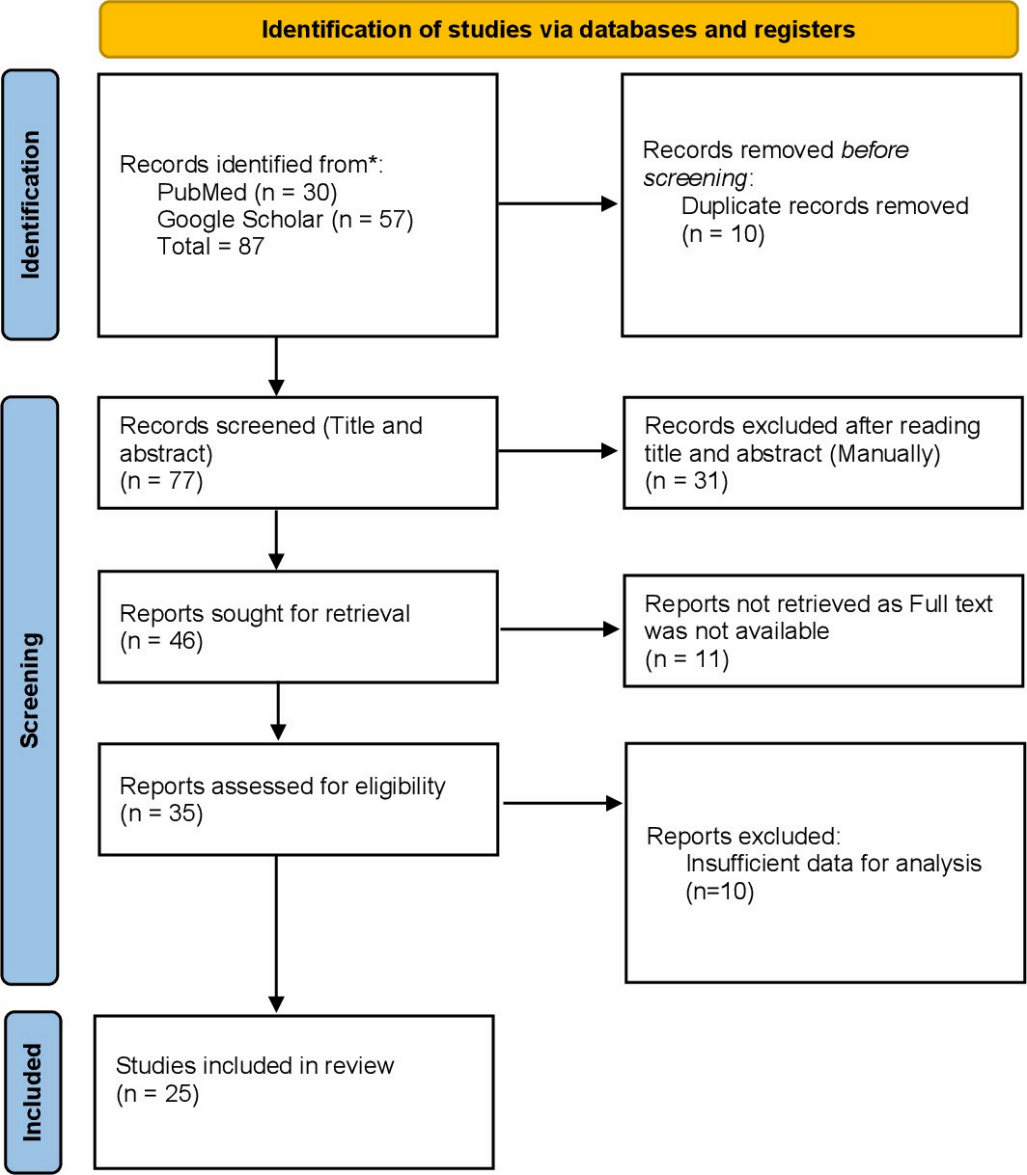
Preferred Reporting Items for Systematic Reviews and Meta-Analyses (PRISMA) diagram detailing the study identification and selection process. *Consider, if feasible to do so, reporting the number of records identified from each database or register searched (rather than the total number across all databases/registers). **If automation tools were used, indicate how many records were excluded by a human and how many were excluded by automation tools. Source: Page MJ, et al. BMJ 2021;372:n71. doi: 10.1136/bmj.n71. This work is licensed under CC BY 4.0. To view a copy of this license, visit https://creativecommons.org/licenses/by/4.0/.

This systematic review was conducted under the guidance of the Preferred Reporting Items for Systematic Reviews and Meta-Analysis (PRISMA) criteria. The Preferred Reporting Items for Systematic Reviews and Meta-Analysis (PRISMA) diagram detailing the selection process is shown in Fig 1. We searched the electronic databases PubMed and Google Scholar and included English-language literature published as full text between April 2015 and August 2024. For PubMed, we limited searches to studies that were relevant to PTSD by applying the Medical Subject Headings (MeSH) Term to “Stress Disorders, Post-Traumatic” or searching for the keywords “stress disorder*” or “PTSD”. For MeSH terms, we used “Earthquakes” for the 2015 earthquake and “Nepal" for Nepal. For the keyword searches, we used “earthquake” for the 2015 earthquake and “Nepal*” for Nepal. The search was restricted to original publications and limited to “Humans” and “English language” and between the time frames of April 2015 and August 2024. For Google Scholar, the keywords PTSD, earthquake, and Nepal were used. The first 5 pages of search results were retrieved. Cross-references from the published articles were manually searched to retrieve additional literature. The authors of some studies were contacted via email and ResearchGate for the retrieval of full texts and the clarification of doubts. The preliminary search strategy is given in the S1 Appendix.

Studies obtained from the electronic databases and manual searches were exported to Google Sheets. Duplicate articles were screened manually. Duplicates were then recorded and removed. After duplicates were removed, the titles and abstracts of the remaining articles were screened independently by two authors (PP, AS). Both authors retrieved the full texts of potentially eligible studies and further screened them for final inclusion. Any disagreement was resolved by consulting with the author (AK) whenever necessary.

### Eligibility criteria

#### Inclusion criteria

Studies eligible for this review had to fulfill the following inclusion criteria: (1) must have been observational and must have assessed PTSD with specific reference to the earthquake; (2) must have examined PTSD diagnosis at least 1 month after the earthquake; (3) must have identified PTSD by established psychiatric interviews according to the Diagnostic and Statistical Manual of Mental Disorders, 5th edition (DSM-V) criteria or the self-reporting questionnaires that were based on the DSM-V; (5) must have included PTSD risk factors selected; and (6) must have defined PTSD with validated scales; and (7) the incidence of PTSD among survivors after earthquakes had to be provided or could be calculated from the data the articles provided.

#### Exclusion criteria

The exclusion criteria were as follows: (1) articles that were not written in English. (2) Articles were reviews, reports, comments or book chapters. (3) Articles contained information that was inaccurate or contradictory. (4) The study population consisted entirely of individuals already suffering from PTSD or from a specific comorbid psychiatric disorder (e.g., depression, attention deficit hyperactivity disorder, substance abuse, or learning disabilities) or having committed a violent offense, which would limit the generalizability of the results. (5) The study contained insufficient data to calculate univariate effect sizes, and such data could not be obtained from the study author.

#### Data abstraction

The literature search was performed by two independent authors (PP, AS), who rigorously screened the titles and abstracts of all the studies identified. Full-text articles were obtained for all the studies satisfying the inclusion criteria. The data extraction was performed independently by the two authors (PP, AS). Any disagreement was resolved by consulting with the author (AK) whenever necessary. For this meta-analysis, the following data were retrieved from the selected articles: Author, year of study, study design, study site, study tool, time of study after the earthquake (in months), sample size, PTSD cases and demographic, traumatic and posttraumatic characteristics as follows:

The demographic characteristics of the participants were as follows: sex (male/female), age group (child <20 years/young and middle-aged adult between 20 and 59 years/old >59 years), marital status (married/unmarried), literacy status (litter/illiterate), and ethnicity status (Brahmin or Chhetri/Janajati/Dalit).

Trauma characteristics: Disease history (yes/no), injury to the body (yes/no), witness death (yes/no), and bereavement (yes/no)

Posttrauma characteristics: Social support (poor/strong), loss of property (yes/no), house damage (yes/no), and involvement in rescue (yes/no)

For each characteristic, the proportions of PTSD cases were tabulated in Google sheets. If, in studies, the proportion of PTSD cases was not analyzed, it was calculated via the available data.

#### Quality evaluation

The quality assessments of each study identified were conducted by two authors (PP and AS) via an 11-item instrument recommended by the Agency for Healthcare Research and Quality (AHRQ) for cross-sectional studies, with eight stars or more considered high quality. Any disagreement was resolved by consulting with the author (AK) whenever necessary. The detailed quality assessment of the articles is shown in S1 Table.

### Statistical analysis

The data collected from the Google Sheets were exported, and analysis was performed via Jamovi 2.3.28 (1). The incidence estimates of PTSD were calculated by pooling the study-specific estimates with 95% confidence intervals via the random effects model by Freeman–Tukey transformation of the inverse hyperbolic sine function. Heterogeneity was evaluated both visually by forest plots and using the χ2 test on Cochrane’s Q statistic and then quantified by calculating the I^2^. The heterogeneity test was considered statistically significant when p ≤ 0.05. In this case, the data were analyzed via a random effects model. In contrast, if p > 0.05, a fixed effects model was used to analyze the data. Subgroup analyses with respect to PTSD incidence by study year, demographic characteristics, trauma characteristics and posttrauma characteristics were performed to explore the possible causes of heterogeneity among studies.

### Sensitivity analyses and publication bias

Sensitivity analyses were performed by omitting one study at a time and calculating the pooled incidence and degree of heterogeneity of the remaining studies to assess whether each individual study influenced the final results [19]. To verify whether publication bias might influence the validity of the incidence, a linear regression method was used, and an Egger funnel plot was then generated. All p values were two-sided, and the cutoff for statistical significance was set at 0.05.

## Results

### Literature search

A total of 87 articles were retrieved via our search strategy, and 10 records were discarded owing to duplication. The titles and abstracts of the remaining 77 articles were screened. We excluded 31 articles and retained the remaining 46 articles for further evaluation by reading the full texts. Eleven reports were not retrieved. Therefore, a total of 35 reports were assessed for eligibility. Among them, 10 reports had insufficient data available and were excluded for that reason [20–29]. Thus, 25 eligible articles were ultimately included in this study.

### Characteristics of eligible articles

The 25 eligible articles considered the 2015 catastrophic earthquake in different districts of Nepal. They analyzed and described the PTSD of the survivors. Only 4 of the 25 eligible articles analyzed and described longitudinal studies, whereas the rest analyzed and described cross-sectional studies. Thirteen eligible articles identified PTSD via the PTSD Checklist (PCL), and 4 articles used the Child PTSD Symptom Scale (CPSS). The detailed characteristics of the included studies are summarized in Table 1.

**Table 1:** Characteristics of the studies included in this systematic review and meta-analysis.

Author	Year	Study design	District	Questionnaire	Time after Earthquake (months)	Sample Size	PTSD Incidence
Acharya et al. [30]	2018	Cross sectional	Kathmandu	CPSS	15	800	409
Jha et al. 1[31]	2017	Longitudinal	Kathmandu	PCL-5	3	333	110
Jha et al. 2[31]	2017	Longitudinal	Kathmandu	PCL-5	11	333	95
Chapagai et al. [32]	2017	Cross sectional	Kathmandu	-	<9	108	9
Sharma et al. [33]	2017	Cross sectional	Kathmandu	-	2	300	30
Mishra et al. [34]	2017	Cross sectional	Kathmandu	PCL-5	<9	140	38
Amgain et al. [35]	2017	Cross sectional	Kavre	PCL-5	-	169	51
Dahal et al. [4]	2018	Cross sectional	Dhading	PCL-C	-	535	99
Kvestad et al. [36]	2019	Cross sectional	Bhaktapur	IES-R	20	552	132
Baral et al. [37]	2019	Cross sectional	Nuwakot	PCL-5	10	291	70
Kane et al. [38]	2018	Cross sectional	Kathmandu, Gorkha, Sindhupalchowk	PCL-C	4	513	27
Schwind et al. 1 [39]	2018	Cross sectional	Gorkha	CPSS	12	62	3
Schwind et al. 2 [40]	2019	Cross sectional	Gorkha	PCL-C	12	238	21
Gautam et al. [41]	2021	Cross sectional	Sindhupalchowk	-	48	331	71
BS et al. [42]	2018	Cross sectional	-	PCL-C	6	305	134
Thapa et al. [43]	2018	Cross sectional	Gorkha	PTSD-8	14	198	53
Pandey et al. [18]	2019	Cross sectional	Gorkha, Dolakha, Sindhupalchowk, Bhaktapur	PCL-C, OSSS-3	36	1076	203
Wagle et al. [44]	2020	Cross sectional	Lalitpur	SQD	>9	362	117
Silwal et al. 1 [45]	2021	Longitudinal	Kathmandu, Sindhupalchowk	CPSS	18	515	134
Silwal et al. 2 [45]	2021	Longitudinal	Kathmandu, Sindhupalchowk	-	31	515	105
Sharma et al. [46]	2019	Cross sectional	Dhading, Chitwan	CPSS	12	409	177
Sharma et al. 2 [47]	2019	Cross sectional	Gorkha	-	-	229	51
Shrestha et al. [48]	2015	Cross sectional	Kathmandu	PCL-5	2	64	11
Thapa et al. [49]	2017	Cross sectional	Bhaktapur	PCL-C	15	289	9
Hatori et al. [50]	2022	Cross sectional	Kathmandu	PCL-5	6	201	32

PCL-5, PTSD Checklist-5; PCL-C, PTSD Checklist-Civilian Version; CPSS, Child PTSD Symptom Scale, IES-R; Impact of Event Scale-Revised, OSSS-3, Oslo Social Support Scale; SQD, Screening Questionnaire for Disaster Mental Health.

### Combined incidence of PTSD after the earthquake

**Fig 2:**
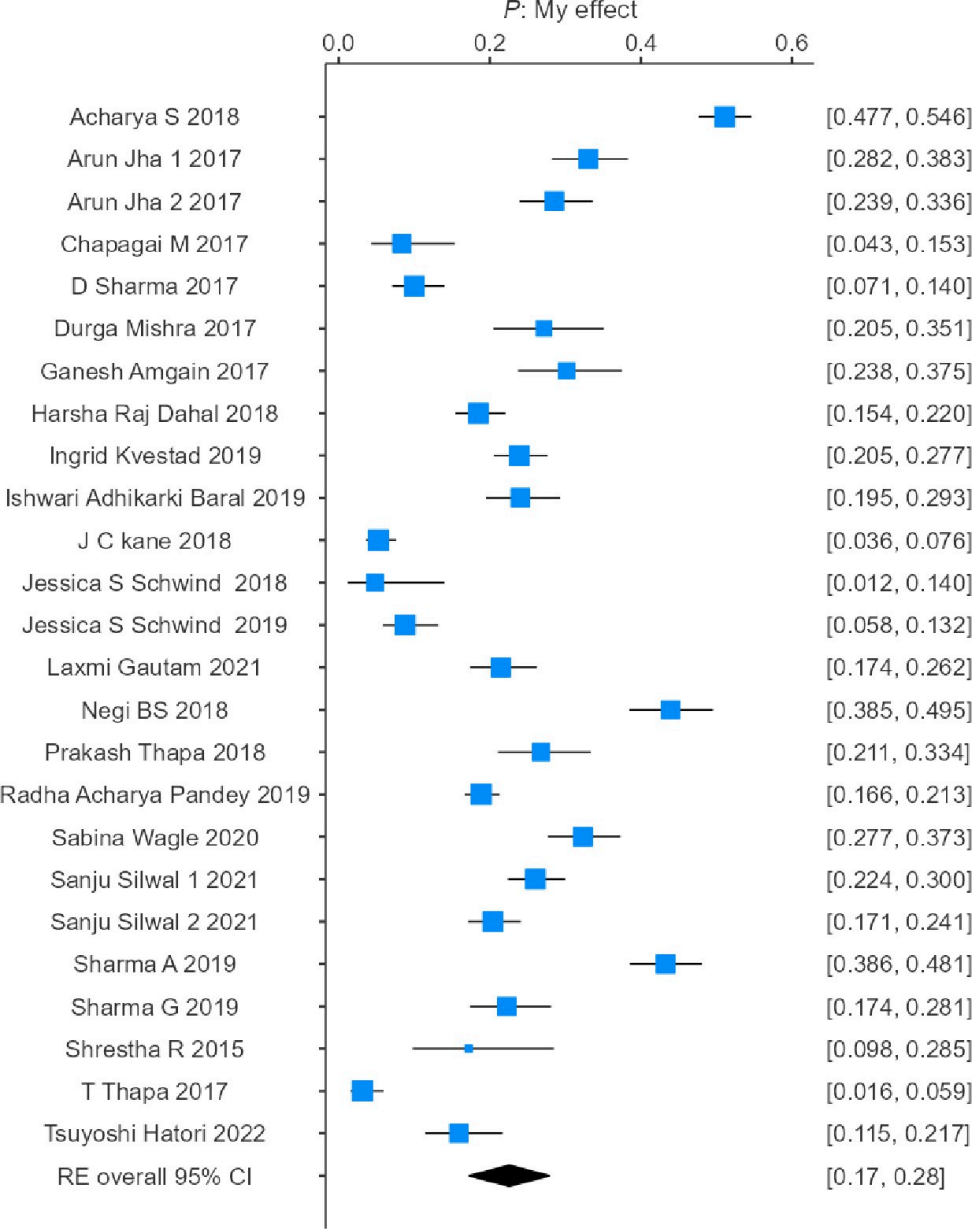
Forest plot showing the estimated pooled incidence of PTSD after the 2015 earthquake in Nepal observed in 25 studies.

A total of 8868 survivors of the earthquake were included in the systematic review and meta-analysis, of which 2191 victims were identified as having PTSD. The heterogeneity test of the eligible studies revealed that they were heterogeneous (I2 = 97.56%; p<0.001). Hence, a random effects model was used to assess the combined incidence of PTSD, which was 22.6% (95% confidence interval: 17.6–27.5%), as represented in the forest plot in Fig 2.

### Sensitivity analysis

Details of the sensitivity analysis are given in S2 Table. These analyses revealed that none of the individual studies influenced the pooled incidence, which ranged from 22.1% (95% CI = 17.1–27.2%) to 23.4% (95% CI = 18.5-]28.2%). There was no significant change in the degree of heterogeneity, which was between 96.91% and 97.72%. The sensitivity analyses indicated that the results of the meta-analysis were reliable and stable.

### Publication bias

**Fig 3:**
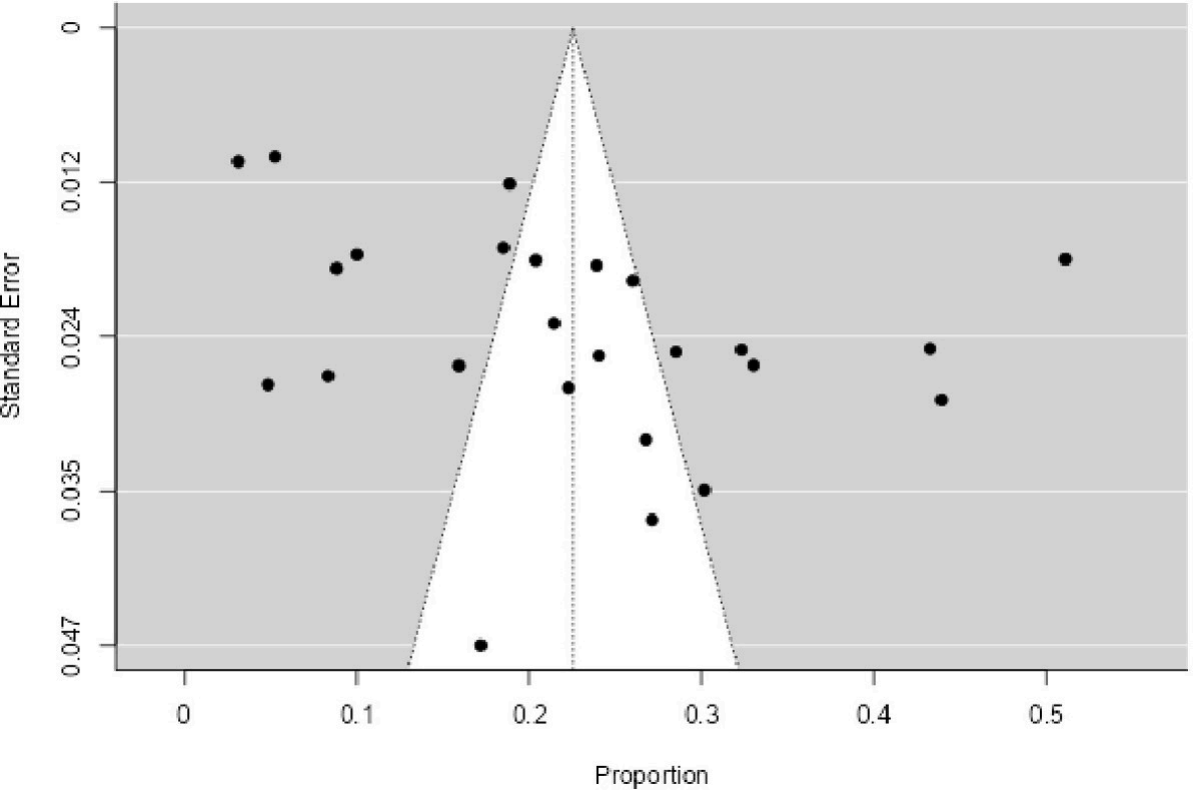
Symmetrical funnel plot of standard error with effect size visualizing 25 studies. (^………^), Pseudo 95% confidence interval; (^…….^), estimated θ_IV_; (.), studies. Random effects p = 0.226. Heterogeneity: τ2 = 0.0177, H2 = 46.2032, I2 = 97.8356%.

Funnel plots of standard errors with effect sizes and linear regression tests for small study effect sizes confirmed that there was no publication bias in any of the 25 included studies reporting the incidence of PTSD after the 2015 earthquake (Eggers’ test: P = 0.192), as shown in Fig 3.

### Subgroup analyses

Subgroup analyses were carried out with respect to PTSD incidence by study year, demographic characteristics, trauma characteristics and posttrauma characteristics. The demographic characteristics included sex, age group, marital status, literacy, and ethnicity. The trauma characteristics included disease history, injury to the body, death witness, and bereavement, and the posttrauma characteristics included social support, loss of property, and damage to the house, which are involved in rescue. Details of subgroup analyses are in Table 2.

**Table 2:** PTSD incidence according to demographic, trauma and posttrauma characteristics.

Study subgroup	Number of Studies	Incidence (95% CI) (%)	Sample	Heterogeneity	P value (Heterogeneity)^a^	P value (Publication Bias)^b^	P value (Interaction)^c^
Total	25	22.6 (17.6–27.5)	8868	97.56	<0.001	0.192	
Demographic Characteristics							
Gender							0.089
Male	14	23.9 (15.6–32.2)	2364	96.32	<0.001	0.459	
Female	14	67.9 (60.3–75.5)	2624	94.7	<0.001	0.879	
Age group							<0.001
Children	3	28.6 (3.7–53.5)	839	92.19	<0.001	<0.001	
Young and Middle Adults	6	20.4 (15.3–25.4)	2219	87.71	<0.001	0.008	
Old	6	27.9 (17–38.7)	372	82.41	<0.001	0.221	
Marital Status							0.482
Married	4	15.5 (8.5–22.5)	1156	83.64	<0.001	0.513	
Unmarried	4	21.1 (9.6–32.5)	650	91.65	<0.001	0.066	
Literacy							<0.001
Literate	4	13.1 (10.2–16) ^d^13.3 (11.6–15.1)	1517	52.65	0.101	0.974	
Illiterate	4	28.8 (24.8–32.7) ^d^28.6 (25.2–32)	677	20.22	0.387	0.489	
Ethnicity							<0.001
Brahmin/Chhetri	3	29.9 (9–50.8)	190	90.9	<0.001	0.817	
Janajati	3	30.2 (3.1–57.4)	428	97.97	<0.001	<0.001	
Dalit	3	34.2 (14.3–54.2)	367	83.24	<0.001	<0.001	
Trauma Characteristics							
Disease History							0.0044
Yes	3	31 (22.1–39.9) ^d^31 (22.1–39.9)	102	0	0.614	0.547	
No	3	20 (12.6–27.3)	685	71.34	0.023	0.866	
Injury to body							<0.001
Yes	4	49.6 (31.2–68)	297	89.05	<0.001	0.15	
No	4	22.9 (10.8–35)	1261	96.64	<0.001	0.278	
Witness Death							<0.001
Yes	3	34.2 (6.3–62.1)	448	97.57	<0.001	0.837	
No	3	7.95 (1.5–14.4)	468	74.53	0.054	0.259	
Bereavement							<0.001
Yes	5	29.6 (12.8–46.3)	340	89.22	<0.001	0.817	
No	5	19.6 (7.1–32.2)	1198	96.94	<0.001	0.888	
Post Trauma Characteristics							
Social Support							<0.001
Poor	3	39 (16.7–61.3)	682	96	<0.001	0.944	
Strong	3	10.3 (3.5–17.1)	1711	93.46	<0.001	0.155	
Loss of Property							<0.001
Yes	4	52 (32.3–71.8)	706	96.66	<0.001	0.063	
No	4	16.6 (3–30.3)	855	96.76	<0.001	0.834	
House Damage							0.07
Yes	4	25.5 (18.4–32.6)	766	79.51	0.002	0.518	
No	4	16.9 (6–27.8)	258	79.33	0.001	0.996	

^a^ P values for heterogeneity across studies were computed via Cochrane’s Q test.

^b^ P values for publication bias were computed via Egger’s regression test.

^c^ P values for comparisons between subgroups were computed via the χ2 test of independence.

^d^ After adjusting for heterogeneity using Fixed-effect model.

**Fig 4:**
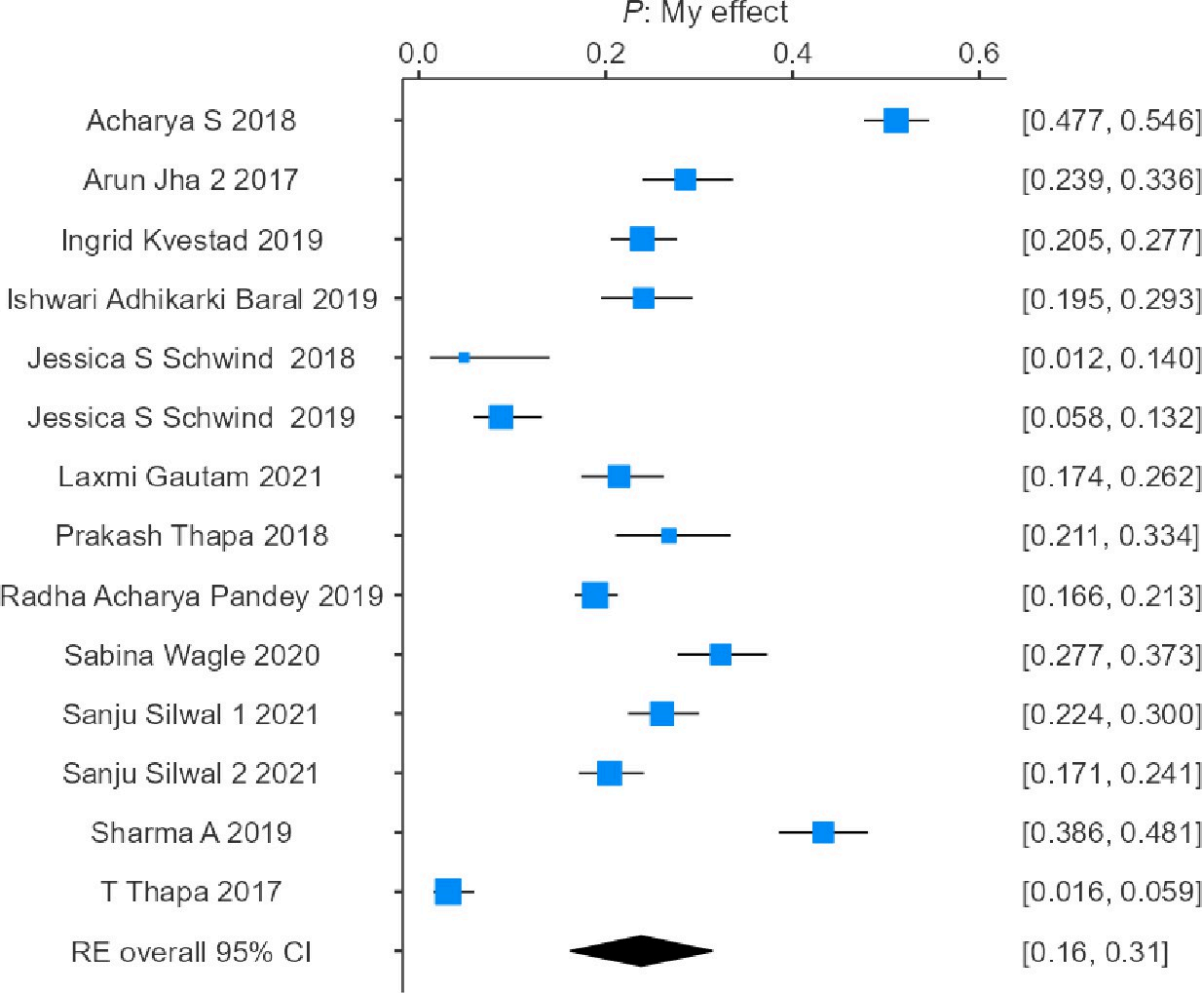
Forest plot showing the estimated pooled incidence of PTSD after the 2015 earthquake in Nepal observed in 8 studies before 9 months.

**Fig 5:**
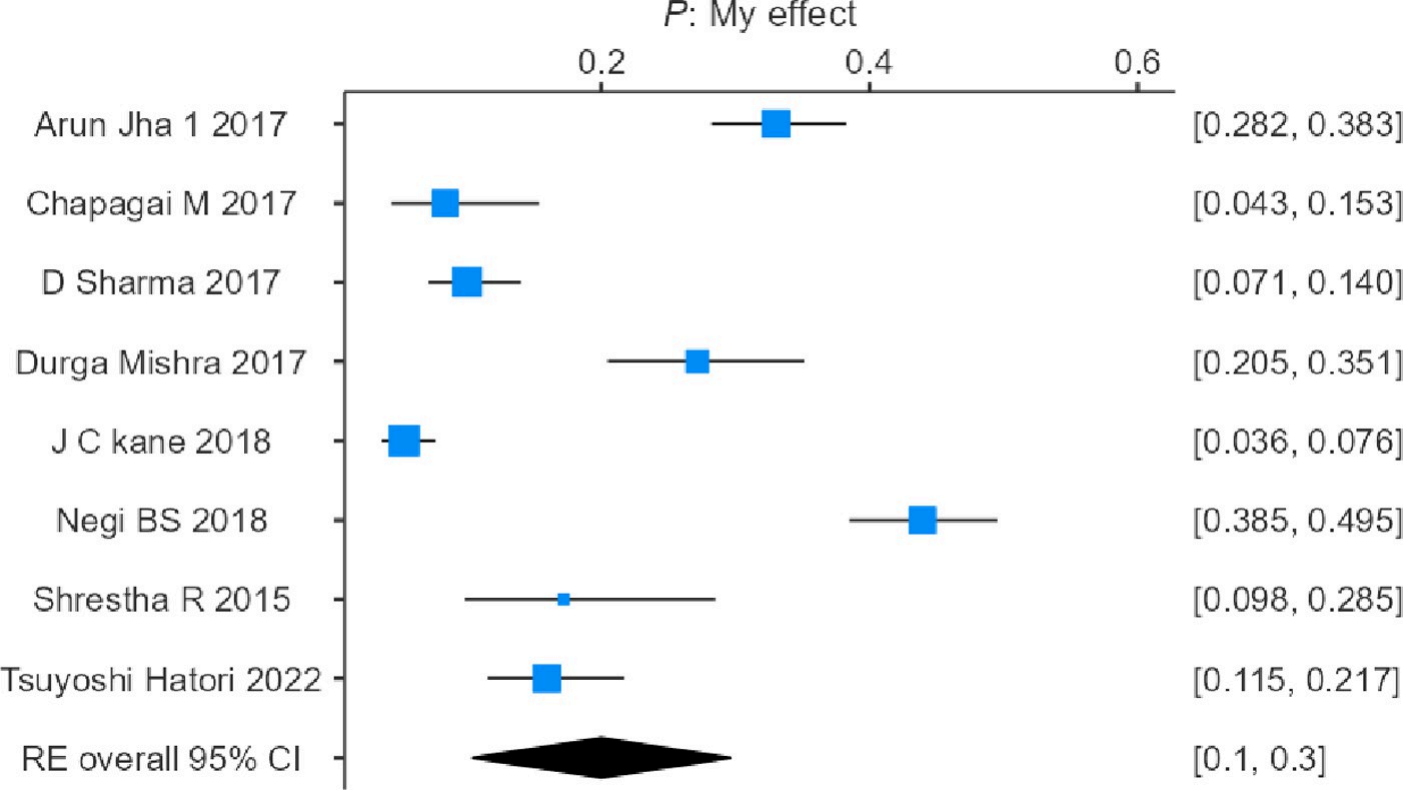
Forest plot showing the estimated pooled incidence of PTSD after the 2015 earthquake in Nepal observed in 14 studies after 9 months.

The results indicated that there was no significant difference in the combined incidence of PTSD among studies with longer follow-up periods (>9 months; combined incidence = 23.8%, 95% CI = 16.3–31.4%; heterogeneity: τ^2^ = 0.0203, H^2^ = 57.6257, I^2^ = 98.2647%) and shorter follow-up periods (≤9 months; combined incidence = 20%, 95% CI = 10.4–29.6%; heterogeneity: τ^2^ = 0.0183, H^2^ = 37.3273, I^2^ = 97.3210%), represented by the forest plots in Figs 4 and 5.

The combined incidence of PTSD among female survivors after earthquakes (67.9%, 95% CI: 60.3–75.5%) was much greater than that among male survivors (23.9%, 95% CI: 15.6–32.2%). Children (combined incidence = 28.6%, 95% CI = 3.7–53.5%), followed by older adults (combined incidence = 27.9%, 95% CI: 17–38.7%), had a higher incidence of PTSD after the 2015 earthquake than young and middle-aged adults did (combined incidence = 20.4%, 95% CI: 15.3–25.4%). In addition, unmarried (21.1%, 95% CI: 9.6–32.5%) and illiterate (adjusted combined incidence = 28.6%, 95% CI: 25.2–32%) survivors were found to have greater incidences of PTSD than literate (adjusted combined incidence = 13.3%, 95% CI: 11.6–15.1%) and married (15.5%, 95% CI: 8.5–22.5%) survivors. Furthermore, the combined incidence of PTSD among Dalit individuals (combined incidence = 34.2%, 95% CI = 14.3–54.2%) was greater than that among Brahmin/Chhetri individuals (combined incidence = 29.9%, 95% CI: 9–50.8%) and Janajati individuals (combined incidence = 30.2%, 95% CI = 3.1–57.4%). Heterogeneity was observed with respect to sex, age group, marital status and ethnicity.

With respect to trauma characteristics, the combined incidence of PTSD among survivors with a disease history (adjusted combined incidence = 31%, 95% CI: 22.1–39.9%) was greater than that among survivors without a disease history (20%, 95% CI: 12.6–27.3%). In addition, the combined incidence of PTSD among survivors with bereavement after an earthquake (29.6%, 95% CI: 12.8–46.3%) was greater than that among survivors without bereavement (19.6%, 95% CI: 7.1–32.2%). Additionally, the combined incidence of PTSD among injured survivors after the earthquake (49.6%, 95% CI: 31.2–68%) was greater than that among non-injured survivors (22.9%, 95% CI: 10.8–35%). Moreover, the combined incidence of PTSD among survivors who had witnessed death after earthquakes (34.2%, 95% CI: 6.3–62.1%) was greater than that among survivors who had not witnessed death (7.95%, 95% CI: 1.5–14.4%). Heterogeneity was observed with respect to injury and bereavement.

Finally, analysis of the post-trauma characteristics of survivors revealed that the combined incidence of PTSD among survivors who had their houses damaged (25.5%, 95% CI: 18.4–32.6%) was greater than that among survivors whose houses were not damaged (16.9%, 95% CI: 6–27.8%). Survivors with loss of property (52%, 95% CI: 32.3–71.8%) had a greater incidence of PTSD than did survivors without loss of property (16.6%, 95% CI: 3–30.3%). Survivors receiving strong social support presented a lower incidence of PTSD (10.3%, 95% CI: 3.5–17.1%) than did those receiving poor social support (39%, 95% CI: 16.7–61.3%). Heterogeneity was observed in each stratum.

Overall, stratification according to these parameters could not entirely explain the heterogeneity of the overall results, with heterogeneity still being high within most subgroups. Furthermore, in subgroup analyses, no publication bias was observed in studies except for those studying the incidence of PTSD among children, Janajati and Dalit, for which the pooled incidence was adjusted via a fixed-effects model.

## Discussion

To the best of our knowledge, this is the first meta-analysis exploring a wide range of risk factors for PTSD among survivors from the 2015 Nepal earthquake, further deepening the understanding of the interaction between demographic and trauma-related and posttrauma factors. While prior studies have examined PTSD in the context of earthquakes, they have often focused on a limited subset of variables, such as physical injuries or loss of property, without integrating broader sociocultural and economic factors unique to disaster-prone regions such as Nepal [17,18]. Given its focus on 12 different risk factors for the prediction of PTSD symptoms, including social support, bereavement, and ethnic differences, this meta-analysis aims to further contextualize existing knowledge on predictors of PTSD. In addition, these methods not only improve generalizations of findings but also fill critically important gaps in the literature that would be useful in helping policy makers and practitioners design better interventions. The findings underline how postdisaster recovery requires a multifaceted strategy addressing not only immediate trauma but also wider socioeconomic and cultural determinants of mental health. This contribution strengthens the evidence base for advocating for the incorporation of mental health considerations into disaster preparedness and response plans in Nepal and other high-risk countries.

The present systematic review and meta-analysis included 8,868 earthquake survivors, of whom 2,191 were diagnosed with PTSD. Owing to considerable heterogeneity among the studies (I² = 97.56%, p<0.001), a random effects model was applied, yielding an overall PTSD incidence of 22.6% (95% CI: 17.6%–27.5%). These findings indicate that approximately 22.6% of earthquake survivors were affected by PTSD. The broad confidence interval observed in this analysis highlights the variability in study outcomes and suggests that factors such as the severity of the earthquake and variations in the availability of support services may influence PTSD rates. This underscores the need for targeted mental health interventions tailored to the specific needs of affected populations.

The results of our analysis are in line with those of a previous meta-analysis by Dai et al. [51], which included forty-six eligible studies encompassing 76,101 earthquake survivors. That study reported a combined PTSD incidence of 28.76% for survivors diagnosed within nine months of the earthquake, compared with 19.48% for those diagnosed more than nine months after the event. A wide-ranging synthesis of the incidence of PTSD after earthquakes worldwide focused on pooled prevalence rates across heterogeneous populations, with no accounting for regional, cultural, or event-specific factors. Although it provides some insights into global trends, it does not delve into the more nuanced impacts of context-specific variables such as socioeconomic vulnerabilities, ethnic disparities, or unique coping mechanisms among affected populations. In contrast, our meta-analysis focuses on survivors of the 2015 Nepal earthquake and presents a comprehensive analysis of 12 risk factors, contextualized to Nepal’s unique sociocultural and economic conditions. This targeted approach provides not only a more accurate picture of PTSD incidence within this disaster-prone region but also bridges the critical gaps in the literature by establishing actionable risk factors that can be used to inform culturally sensitive and region-specific mental health interventions.

Moreover, a meta-analysis by Hosseinnejad et al. (2022) on the prevalence of PTSD following earthquakes in Iran and Pakistan reported a pooled prevalence of 55.6%, [52] which is notably higher than the results of the current analysis.

According to the analysis, the most significant risk factor for the development of PTSD is sex. This finding is consistent with a cross-sectional study that also revealed that the likelihood of developing PTSD was 1.6 times greater in women than in men [3]. Similarly, as per the article of Anderson and Manuel [53], in response to the earthquake, women reported feeling more stressed. The gender disparity in the incidence of PTSD, with significantly higher rates among women than among men, is a well-documented phenomenon observed across cultures and disasters [17,53]. While biological factors, such as heightened stress reactivity and differences in the HPA axis, partially explain this pattern, social and cultural dynamics in Nepal further amplify these disparities. Traditional gender roles place disproportionate caregiving and emotional burdens on women during crises, whereas societal norms often restrict their access to resources and mental health services.

Women in Nepal’s patriarchic context are often restricted in mobility and decision-making, which may delay help-seeking and increase stress. Moreover, the socialization of gender roles encourages emotional expressivity among women, leading them to report distress, whereas men might suppress symptoms, thereby underestimating their psychological burden [46]. These biological and sociocultural factors intersect to highlight the urgent need for gender-sensitive mental health interventions fitted to the Nepalese context, with equal access to care and systemic barriers.

In addition, our results suggest that after an earthquake, children are more likely to develop PTSD than young and middle-aged adults and older people. According to Yuwei Li [54], several factors, including feeling that one’s own or a family member’s life is in danger, losing a close friend or family member, extraversion, neuroticism, TrkB, G72, and CNTF, have been linked to the maintenance of PTSD symptoms.

Yong Liang reported that, in the elderly population, the overall occurrence of PTSD after earthquakes was 25% [55]. Our study revealed that the incidence of PTSD among earthquake survivors of elderly age (27.9%) was slightly greater than that reported among earthquake survivors in China.

According to several earlier studies [56], subgroup analyses also suggested that witnessing death, bereavement, physical harm, and property damage all contribute to various PTSD incidences. This means that people who had witnessed death, experienced bereavement, or suffered more personal injury or property loss were more likely to develop PTSD [57]. Those who are illiterate and female have a greater risk of developing PTSD. These results align with the findings of numerous disaster psychology studies [57,58].

The majority of the eligible studies, according to the quality evaluation, lacked sufficient subgroup analyses; as a result, many of the variables included in the analyses were only examined in a small proportion of studies, which limits the conclusions that can be drawn. Furthermore, they detected PTSD via self-report questionnaires, which warrants that the overall results be interpreted with caution, as the combined incidence of PTSD might be overestimated. Although many possible risk factors from the eligible articles were extracted, a high degree of heterogeneity was detected when the combined incidence was analyzed and subgroup analyses were conducted. Owing to differences in sampling, design, measurement, and statistical analysis, this is not uncommon in reviews of observational studies [59]. Our study, however, highlights areas that warrant further investigations, such as a detailed analysis of risk factors for posttraumatic stress disorder after the 2015 earthquake in Nepal.

Natural disasters are common in Nepal. Numerous such tragic events take place each year, but few studies have examined and explained survivors’ psychological states. Their neglected mental state caused them to suffer all of their lives, which has a significant effect on society’s peace and prosperity. The government of Nepal could benefit from some information from this meta-analysis about the incidence of mental health issues and associated risk factors, which would enable them to emphasize psychological first aid in the aftermath of disasters such as earthquakes and allocate resources accordingly. In a similar vein, this paper may serve as a reference for future studies on the stress experienced by earthquake survivors in 2015, providing an international perspective on the conditions surrounding the development of mental health issues in the Nepali community after a disaster.

## Supporting information

S1 Appendix Search strategy used in the current systematic review and meta- analysis. (DOCX)

S2 Appendix Risk of Bias Assessment using the Agency for Healthcare Research and Quality (AHRQ) checklist. (DOCX)

S3 Appendix Data Set. (XLSX)

S1 Table Quality assessment of the included articles using the Agency for Healthcare Research and Quality (AHRQ) checklist. (DOCX)

S2 Table Sensitivity analysis by omitting one study at a time via a random effects model. (DOCX)

S3 Table PRISMA 2020 item checklist. (DOCX)

S4 Table PRISMA 2020 abstracts checklist. (DOCX)
