# Supplementary material for: Posttraumatic stress disorder and associated factors in the aftermath of the 2015 earthquake in Nepal: A systematic review and meta-analysis
Source: PLoS One. 2025 Feb 3;20(2):e0310233. doi: 10.1371/journal.pone.0310233 (PMC11790126; doi:10.1371/journal.pone.0310233)
Supplement: S1 Appendix — (DOCX) [file pone.0310233.s001.docx]

**S1 Appendix. Search strategy used in the current systematic review and meta- analysis.**

For PubMed

#1: "stress disorders, post traumatic"[MeSH Terms]

#2: "stress disorder*"[tiab] OR PTSD[tiab]

#3: #1 OR #2

#4: "Earthquakes"[Mesh]

#5: “Earthquake*”[tiab]

#6: #4 OR #5

#7: “Nepal”[Mesh]

#8: “Nepal”[tiab]

#9: #7 OR #8

#10: #3 AND #6 AND #9

Filters: Human Subject, English, Time frame from 2015 to 2024

Final Search Strategy after filters:

(("stress disorders, post traumatic"[MeSH Terms] OR ("stress disorder*"[Title/Abstract] OR "PTSD"[Title/Abstract])) AND ("Earthquakes"[MeSH Terms] OR "earthquake*"[Title/Abstract]) AND ("nepal"[MeSH Terms] OR "nepal*"[Title/Abstract])) AND ((humans[Filter]) AND (2015:2024[pdat]) AND (english[Filter]))
