# Supplementary material for: Posttraumatic stress disorder and associated factors in the aftermath of the 2015 earthquake in Nepal: A systematic review and meta-analysis
Source: PLoS One. 2025 Feb 3;20(2):e0310233. doi: 10.1371/journal.pone.0310233 (PMC11790126; doi:10.1371/journal.pone.0310233)
Supplement: S2 Appendix — (DOCX) [file pone.0310233.s002.docx]

**S2 Appendix. Risk of Bias Assessment using the Agency for Healthcare Research and Quality (AHRQ) Checklist**

1. Define the source of information (survey, record review)

2. List inclusion and exclusion criteria for exposed and unexposed subjects (cases and controls) or refer to previous publication

3. Indicate time period used for identifying patients

4. Indicate whether or not subjects were consecutive if not population-based

5. Indicate if evaluators of subjective components of study were masked to other aspects of the status of participants

6. Describe any assessments undertaken for quality assurance purposes

7. Explain any patient exclusions from analysis

8. Describe how confounding was assessed and/or controlled

9. If applicable, explain how missing data were handled in the analysis

10. Summarize patient response rates and completeness of data collection

11. Clarify what follow-up, if any was expected and the percentage of patients for which incomplete data or follow-up was obtained.
