# Supplementary material for: Posttraumatic stress disorder and associated factors in the aftermath of the 2015 earthquake in Nepal: A systematic review and meta-analysis
Source: PLoS One. 2025 Feb 3;20(2):e0310233. doi: 10.1371/journal.pone.0310233 (PMC11790126; doi:10.1371/journal.pone.0310233)
Supplement: S2 Table — (DOCX) [file pone.0310233.s005.docx]

**S2 Table. Sensitivity Analysis by omitting one study at a time using random effect model**

| Author Year | Incidence (%) | Lower 95% C.I. | Upper 95% C.I. | Tau^2^ | I^2^ (%) |
| --- | --- | --- | --- | --- | --- |
| Omitting Acharya S 2018[1] | 21.3 | 16.8 | 25.8 | 0.0119 | 96.91 |
| Omitting Arun Jha 1 2017[2] | 22.1 | 17.1 | 27.2 | 0.0153 | 97.63 |
| Omitting Arun Jha 2 2017[2] | 22.3 | 17.2 | 27.4 | 0.0156 | 97.67 |
| Omitting Chapagai M 2017[3] | 23.1 | 18.2 | 28.1 | 0.0149 | 97.57 |
| Omitting D Sharma 2017[4] | 23.1 | 18.1 | 28.1 | 0.015 | 97.52 |
| Omitting Durga Mishra 2017[5] | 22.4 | 17.3 | 27.5 | 0.0156 | 97.71 |
| Omitting Ganesh Amgain 2017[6] | 22.2 | 17.2 | 27.3 | 0.0155 | 97.69 |
| Omitting Harsha Raj Dahal 2018[7] | 22.7 | 17.6 | 27.8 | 0.0157 | 97.62 |
| Omitting Ingrid Kvestad 2019[8] | 22.5 | 17.4 | 27.6 | 0.0158 | 97.64 |
| Omitting Ishwari Adhikarki Baral 2019[9] | 22.5 | 17.4 | 27.6 | 0.0157 | 97.7 |
| Omitting J C kane 2018[10] | 23.3 | 18.4 | 28.2 | 0.0144 | 97.13 |
| Omitting Jessica S Schwind 2018[11] | 23.3 | 18.4 | 28.2 | 0.0144 | 97.5 |
| Omitting Jessica S Schwind 2019[11] | 23.1 | 18.1 | 28.1 | 0.0149 | 97.52 |
| Omitting Laxmi Gautam 2021[12] | 22.6 | 17.5 | 27.7 | 0.0158 | 97.68 |
| Omitting Negi BS 2018[13] | 21.7 | 16.9 | 26.5 | 0.0138 | 97.39 |
| Omitting Prakash Thapa 2018[14] | 22.4 | 17.3 | 27.5 | 0.0157 | 97.71 |
| Omitting Radha Acharya Pandey 2019[15] | 22.7 | 17.6 | 27.8 | 0.0157 | 97.49 |
| Omitting Sabina Wagle 2020[16] | 22.1 | 17.1 | 27.2 | 0.0153 | 97.63 |
| Omitting Sanju Silwal 1 2021[17] | 22.4 | 17.3 | 27.5 | 0.0157 | 97.65 |
| Omitting Sanju Silwal 2 2021[17] | 22.6 | 17.5 | 27.8 | 0.0158 | 97.64 |
| Omitting Sharma A 2019[18] | 21.7 | 16.9 | 26.5 | 0.0138 | 97.38 |
| Omitting Sharma G 2019[19] | 22.6 | 17.4 | 27.7 | 0.0158 | 97.71 |
| Omitting Shrestha R 2015[20] | 22.8 | 17.7 | 27.8 | 0.0156 | 97.72 |
| Omitting T Thapa 2017[21] | 23.4 | 18.5 | 28.2 | 0.014 | 97.09 |
| Omitting Tsuyoshi Hatori 2022[22] | 22.8 | 17.7 | 27.9 | 0.0156 | 97.67 |
| Total | 22.6 | 17.6 | 27.5 | 0.0151 | 97.56 |

References for S2 Table

[1] Acharya S, Bhatta DN, Assannangkornchai S. Post-Traumatic Stress Disorder Symptoms Among Children of Kathmandu 1 Year After the 2015 Earthquake in Nepal. Disaster Med Public Health Prep 2018;12:486–92. https://doi.org/DOI: 10.1017/dmp.2017.100.

[2] Jha A, Shakya S, Zang Y, Pathak N, Pradhan P, Bhatta K, et al. Identification and treatment of Nepal 2015 earthquake survivors with posttraumatic stress disorder by nonspecialist volunteers: An exploratory cross-sectional study. Indian J Psychiatry 2017;59:320. https://doi.org/10.4103/psychiatry.IndianJPsychiatry_236_16.

[3] Chapagai M, Tulachan P, Shakya S, Dhungana S, Pant S. Psychiatric morbidity pattern in patient after earthquake at Tribhuvan University Teaching Hospital Nepal. Journal of Institute of Medicine Nepal 2017;39:16–20. https://doi.org/10.59779/jiomnepal.821.

[4] Sharma D, Devi A, Rc K. Mental Health Impact of April 2015 Earthquake of Nepal : A Community Based Cross-sectional Study. 2017.

[5] Mishra D, Giri D, Marahatta SB. Post-Traumatic Stress Disorder and associated factors among adolescents after 2015 Nepal Earthquake. Journal of Manmohan Memorial Institute of Health Sciences 2018;3:45–55. https://doi.org/10.3126/jmmihs.v3i1.19178.

[6] Amgain G, Kapil Amgain D, Neupane S. PTSD and its influencing factors among the government school teachers after Nepal Earthquake 2015. Elixir 2017:49265–8.

[7] Dahal HR, Kumar S, Thapa DK. Prevalence and risk factors of post-traumatic stress disorders among the survivors of 2015 Nepal earthquake, in Dhading, Nepal. Sleep and Hypnosis 2018;20:128–39. https://doi.org/10.5350/Sleep.Hypn.2017.19.0145.

[8] Kvestad I, Ranjitkar S, Ulak M, Chandyo RK, Shrestha M, Shrestha L, et al. Earthquake Exposure and Post-traumatic Stress Among Nepalese Mothers After the 2015 Earthquakes. Front Psychol 2019;10. https://doi.org/10.3389/fpsyg.2019.00734.

[9] Adhikari Baral I, K.C B. Post traumatic stress disorder and coping strategies among adult survivors of earthquake, Nepal. BMC Psychiatry 2019;19:118. https://doi.org/10.1186/s12888-019-2090-y.

[10] Kane JC, Luitel NP, Jordans MJD, Kohrt BA, Weissbecker I, Tol WA. Mental health and psychosocial problems in the aftermath of the Nepal earthquakes: findings from a representative cluster sample survey. Epidemiol Psychiatr Sci 2018;27:301–10. https://doi.org/DOI: 10.1017/S2045796016001104.

[11] Schwind JS, Norman SA, Brown R, Frances RH, Koss E, Karmacharya D, et al. Association Between Earthquake Exposures and Mental Health Outcomes in Phulpingdanda Village After the 2015 Nepal Earthquakes. Community Ment Health J 2019;55:1103–13. https://doi.org/10.1007/s10597-019-00404-w.

[12] Gautam L, Mishra DK, Pant GP, Khadka R, Chataut PD, Shrestha T, et al. Long term post-traumatic stress disorders among the earthquake affected people of Sindhupalchwok, Nepal. Int J Community Med Public Health 2021;8:5643. https://doi.org/10.18203/2394-6040.ijcmph20214550.

[13] Negi BS, Joshi SK, Nakazawa M, Kotaki T, Bastola A, Kameoka M. Impact of a massive earthquake on adherence to antiretroviral therapy, mental health, and treatment failure among people living with HIV in Nepal. PLoS One 2018;13:e0198071. https://doi.org/10.1371/journal.pone.0198071.

[14] Thapa P, Acharya L, Dev Bhatta B, Paneru SB, Khattri JB, Chakraborty PK, et al. Anxiety, Depression and Post-Traumatic Stress Disorder after Earthquake. vol. 16. 2018.

[15] Acharya Pandey R, Chalise P, Khadka S, Chaulagain B, Maharjan B, Pandey J, et al. Post-traumatic stress disorder and its associated factors among survivors of 2015 earthquake in Nepal. BMC Psychiatry 2023;23:340. https://doi.org/10.1186/s12888-023-04836-3.

[16] Wagle S, Amnatsatsue K, Adhikari B, Kerdmongkol P, Van der Putten M, Silpasuwan P. Health-Related Quality of Life After the 2015 Gorkha Earthquakes, Among Older Adults Living in Lalitpur District of Central Nepal. Disaster Med Public Health Prep 2021;15:298–307. https://doi.org/DOI: 10.1017/dmp.2019.154.

[17] Silwal S, Dybdahl R, Chudal R, Sourander A, Lien L. Psychiatric symptoms experienced by adolescents in Nepal following the 2015 earthquakes. J Affect Disord 2018;234:239–46. https://doi.org/10.1016/j.jad.2018.03.002.

[18] Sharma A, Kar N. Posttraumatic Stress, Depression, and Coping Following the 2015 Nepal Earthquake: A Study on Adolescents. Disaster Med Public Health Prep 2019;13:236–42. https://doi.org/DOI: 10.1017/dmp.2018.37.

[19] Sharma G, Sapkota B, Sharma G, Paudel S, Lamichhane G, Adhikari M, et al. MENTAL HEALTH IMPACT OF NEPAL EARTHQUAKE 2015 ON THE POPULATION OF GORKHA DISTRICT 2018.

[20] Shrestha R. Post-traumatic Stress Disorder among Medical Personnel after Nepal earthquake, 2015. vol. 13. n.d.

[21] Thapa T, Hawlader M, Hossain M, Chowdhury A, Nabi M, Zaman S, et al. Status of Post-traumatic Stress Disorder (PTSD) and Its Associated Factors among Secondary School Students after Fifteen Months of Earthquake in Bhaktapur District, Nepal. Asian Journal of Medicine and Health 2017;7:1–9. https://doi.org/10.9734/AJMAH/2017/36819.

[22] Hatori T, Bhandary NP. Posttraumatic stress disorder and its predictors in Kathmandu Valley residents after the 2015 Nepal Earthquake. International Journal of Disaster Risk Reduction 2022;69:102733. https://doi.org/10.1016/j.ijdrr.2021.102733.
