# Supplementary material for: Posttraumatic stress disorder and associated factors in the aftermath of the 2015 earthquake in Nepal: A systematic review and meta-analysis
Source: PLoS One. 2025 Feb 3;20(2):e0310233. doi: 10.1371/journal.pone.0310233 (PMC11790126; doi:10.1371/journal.pone.0310233)
Supplement: S4 Table — (DOCX) [file pone.0310233.s007.docx]

**S4 Table.** **PRISMA 2020 for Abstracts checklist.**

| **Section and Topic** | **Item #** | **Checklist item** | **Reported on page #** |
| --- | --- | --- | --- |
| **TITLE** | | |  |
| Title | 1 | Identify the report as a systematic review. | I |
| **Background** | | |  |
| Objectives | 2 | Provide an explicit statement of the main objective(s) or question(s) the review addresses. | 1 |
| **METHODS** | | |  |
| Eligibility criteria | 3 | Specify the inclusion and exclusion criteria for the review. | 1 |
| Information sources | 4 | Specify the information sources (e.g. databases, registers) used to identify studies and the date when each was last searched. | 1 |
| Risk of bias |  | Specify the methods used to assess risk of bias in the included studies. | 1 |
| Synthesis of results |  | Specify the methods used to present and synthesise results. | 1 |
| **RESULTS** | | | |
| Included studies | 3 | Give the total number of included studies and participants and summarise relevant characteristics of studies. | 1 |
| Synthesis of results | 4 | Present results for main outcomes, preferably indicating the number of included studies and participants for each. If meta-analysis was done, report the summary estimate and confidence/credible interval. If comparing groups, indicate the direction of the effect (i.e. which group is favoured). | 1 |
| **DISCUSSION** | | | |
| Limitations of evidence |  | Provide a brief summary of the limitations of the evidence included in the review (e.g. study risk of bias, inconsistency and imprecision). | 1 |
| Interpretation |  | Provide a general interpretation of the results and important implications | 2 |
| **OTHER** | | | |
| Funding |  | Specify the primary source of funding for the review. | N/A |
| Registration |  | Provide the register name and registration number. | 1 |

*From:*  Page MJ, McKenzie JE, Bossuyt PM, Boutron I, Hoffmann TC, Mulrow CD, et al. The PRISMA 2020 statement: an updated guideline for reporting systematic reviews. BMJ 2021;372:n71. doi: 10.1136/bmj.n71
